# Emission efficiency limit of Si nanocrystals

**DOI:** 10.1038/srep19566

**Published:** 2016-01-20

**Authors:** Rens Limpens, Stefan L. Luxembourg, Arthur W. Weeber, Tom Gregorkiewicz

**Affiliations:** 1 Van der Waals-Zeeman Institute, University of Amsterdam, 1098 XH Amsterdam, The Netherlands; 2 ECN Solar Energy, PO Box 1, 1755 ZG Petten, The Netherlands; 3 Photovoltaic Materials and Devices, Delft University of Technology, 2600 GA Delft, The Netherlands

## Abstract

One of the important obstacles on the way to application of Si nanocrystals for development of practical devices is their typically low emissivity. In this study we explore the limits of external quantum yield of photoluminescence of solid-state dispersions of Si nanocrystals in SiO_2_. By making use of a low-temperature hydrogen passivation treatment we demonstrate a maximum emission quantum efficiency of approximately 35%. This is the highest value ever reported for this type of material. By cross-correlating PL lifetime with EQE values, we obtain a comprehensive understanding of the efficiency limiting processes induced by P_b_-defects. We establish that the observed record efficiency corresponds to an interface density of P_b_-centers of 1.3 × 10^12^ cm^12^, which is 2 orders of magnitude higher than for the best Si/SiO_2_ interface. This result implies that Si nanocrystals with up to 100% emission efficiency are feasible.

Semiconductor nanocrystals (NCs) feature a lot of attractive properties, due to a combination of quantum confinement and surface prevalence but their wide-spread application in practical devices is yet to appear. This is due to the fact that the NCs with the best optical characteristics contain either scarce (In, Ga) or toxic (Cd, As) elements, while the emissivity of Si NCs – the most obvious sustainable alternative – remains low. Here, we investigate the natural emissivity efficiency limit of the basic, and so far only truly up-scalable form of Si NC networks: thin-film solid-state dispersions of Si NCs embedded in SiO_2_. In this way we establish the true application potential of this material.

In general, it can be expected that the smaller the Si NC size, the better the optical quality, since relaxation of momentum conservation and increased Coulombic interactions boost the optical transition rate of this indirect bandgap material^1^. On the other hand, for smaller NCs, the crystallographic quality of their surface deteriorates due to the increased curvature, thus decreasing the optical yield^2^. As a matter of fact, it is well known that the emission efficiency of Si NCs depends crucially on the surface quality^3^ and relatively high emission efficiencies have been reported for colloidal Si NCs with specifically engineered surfaces^4^. Specifically for the Si/SiO_2_ interface, it has been shown that P_*b*_ defects are present^5^, featuring unpaired electrons (“dangling bonds”) which function as efficient centers of nonradiative recombination^6^,^7^,^8^. Here, we apply low-temperature annealing in hydrogen (H_2_) as passivation treatment, to de-activate the P_b_-centers (known for its efficient passivation^9^), and investigate the emissivity limit of SiO_2_ embedded Si NCs. We demonstrate a maximum EQE of approximately 35%. To our knowledge, this is the highest EQE ever reported for this type of material; it is obtained for NC sizes between 3–4 nm. According to the model we present in this paper, the current maximum EQE corresponds to an interface density of P_b_-centers of [P_b_] ≈ 1.3 × 10^12^ cm^−2^. By cross-correlating PL lifetime with EQE, we conclude that energy transfer towards “defected” NCs containing a P_b_-center strongly reduces the internal quantum efficiencies.

## Sample Preparation and Characterization

In this investigation, we make use of Si NCs in SiO_2_, prepared by sputtering and a high-temperature heat treatment (at 1100, 1150 and 1200 °C) to induce Si precipitation and NC formation – see Experimental Methods for more material details. Subsequently, some of the samples have been passivated by a 2-hour annealing at a temperature of 500 °C in H_2_-atmosphere – this treatment was optimized to reduce the concentration of P_b_-centers (Supplementary Information) and improve the optical quality of Si NCs.

**Figure 1:**
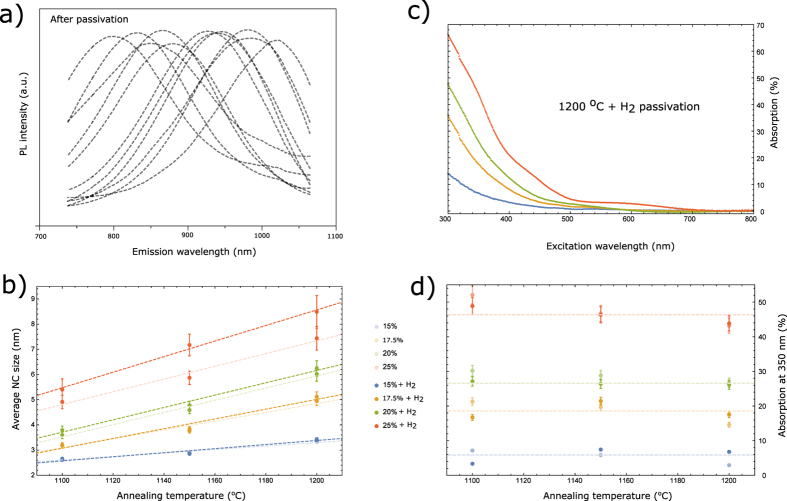
Optical characterization. (**a**) Normalized PL emission spectra of all surface passivated Si NC ensembles, at an excitation wavelength of 400 nm. (**b**) Average NC sizes for all combinations of production parameters (formation temperature, passivation and Si content), derived from^10^. (**c**) Absorption spectra for samples with different Si contents while having similar annealing conditions. This behavior is illustrative for all spectra. (**d**) Absorption values at an excitation wavelength of 350 nm for all combinations of production parameters (formation temperature, passivation and Si content).

In Fig. 1a we depict emission spectra of the passivated samples and in Fig. 1b the average NC sizes before and after passivation, as a function of annealing temperature, for different amounts of excess Si. The sizes have been deduced from the PL spectra following Takeoka *et al.*^10^. Similar to previous reports^11^, the average size of NCs increases with the annealing temperature and the Si excess, but also seems to increase upon passivation, with the change being more pronounced for ensembles with a larger average NC size.

In Fig. 1c we present typical absorption spectra and in Fig. 1d, the absorption values for the investigated samples at an excitation wavelength of 350 nm. Clearly, the absorption follows the amount of excess Si, being practically independent on the annealing conditions. We note that the changes in the NC size distribution are not being reflected in the absorption features. Further we observe that the passivation procedure has no effect on the absorption.

## Results and Interpretation

We start by commenting on an apparent modification of the average NC size appearing upon annealing - Fig. 1b. Since the passivation treatment is performed at temperatures below those necessary to alter the Si aggregation process^12^, this effect cannot originate from an actual change of the NC size. To understand the experimental results of Fig. 1b we recall that the depicted NC size is actually derived from the PL spectrum, and thus the origin of its apparent increase is a red-shift of the ensemble PL spectrum upon passivation treatment. This is readily explained by the passivation effect having a greater impact on the population of larger NCs, whose probability of being defected is higher due to their larger surface area^13^. Therefore, the present experiment evidences that the ensemble of optically active (not “defective”) NCs contribution to PL spectrum is modified by the H_2_ passivation treatment – as suggested by^11^.

Next, from the absorption data presented in Fig. 1d, we ascribe the apparent invariance of absorption to the NC size distribution and passivation, to the indirect band-structure of the Si NCs. Where emission proceeds via band-edge states (which are highly sensitive to the NC size, as observed in Fig. 1b), absorption takes place via states that are positioned higher in the bands as well, and these are much less influenced by quantum confinement.

Subsequently, we correlate changes of dynamics and EQE^4^ of excitonic PL upon passivation treatment. We note that the fast non-radiative recombination induced by the P_b_-centers^14^, cannot be revealed in our PL dynamics, since the time-resolution of the experimental setup is limited by the laser pulse duration to approximately 10 ns^9^). In contrast, the EQE measurements reflect the ratio of emitted and absorbed photons and therefore do reveal the presence of defected NCs, which contribute to absorption but not to emission. Hence, one can expect that the “defect healing” action of the H_2_ passivation treatment will not change PL dynamics and will result in an increase of the EQE. However, we will show that the cross-correlation of PL dynamics and EQE measured for the same materials allows for unique insights into the quenching mechanisms of excitonic PL of Si NCs.

**Figure 2:**
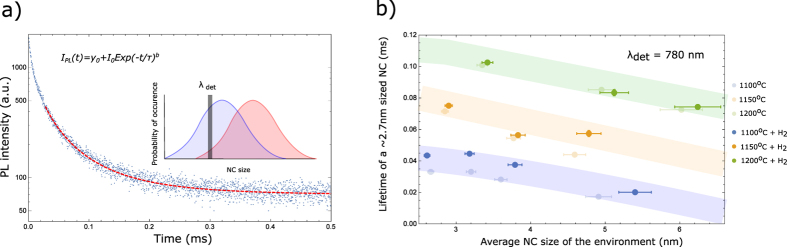
Effective lifetimes. (**a**) Stretched exponential fit of a typical transient. The performed measurements are schematically illustrated in the inset. Measuring several NC ensembles at a fixed detection wavelength allows to monitor the impact of the ensemble on the excitonic emission of a certain sized NC (**b**) Effective lifetimes of a 2.7 sized NC, as a function on the environment NC size, before and after passivation. An excitation wavelength of 520 nm is used. The environment is varied by changing the formation temperature and Si content. The colored shades indicate the NC-size (of the environment) dependent lifetimes.

In Fig. 2b, we depict the effective PL decay times measured for different samples at a detection wavelength of 780 nm (a typical transient is shown in Fig. 2a). Fixing the detection wavelength allows to monitor excitonic emission from NCs of a certain size (here around 2.7 nm in diameter) within different ensembles of which the average sizes were already depicted in Fig. 1b. Due to the large number of samples investigated in this study, certain trends with respect to the NC size can be deduced (indicated by the colored lines). From the data in Fig. 2, the following observations can be made:

1. For samples prepared under the same annealing conditions, the effective lifetime decreases as the average NC size of the ensemble increases, i.e. for a larger Si excess.
2. For samples with the same NC environment, the effective lifetime increases for higher formation temperatures.
3. At variance with the expectation, H_2_-passivation increases the effective lifetime, with the change being more pronounced for samples prepared at lower formation temperatures.

Under assumption that the P_b_-centers facilitate the dominant quenching mechanism of excitonic PL, passivation cannot induce detectable changes of the effective lifetime. Therefore the experimental results indicate the presence of yet another channel of non-radiative recombination which is also affected by the applied treatment. In fact, in our previous investigations of energy transfer within ensembles of Si NCs^15^, we have demonstrated that energy transfer towards non-emitting NCs effectively functions as an additional path of relatively slow non-radiative recombination. Now we argue that the experimental data of Fig. 2 are readily explained by the same mechanism. Since we make use of a narrow detection wavelength window, the energy transfer towards a larger NC (emitting outside of the detection range) results in PL intensity loss, detected as reduction of the effective lifetimes – similar to the transfer towards defected NCs. This lifetime quenching will be stronger for ensembles with a larger average NC size, due to a more pronounced excitation diffusion (observation #1). By increasing the formation temperature, the P_b_-defect density is reduced^16^ and, as a result, the energy migration path is extended. This leads to an increase in the effective lifetime (observation #2). Similarly, the P_b_-center density is also reduced by the H_2_ passivation^9^. This effect is stronger in materials with initially a higher defect density, i.e. those developed at lower formation temperatures (observation #3). It should be noted that this quenching model of P_b_-centers in combination with energy migration is able to explain variations in internal quantum efficiencies for different Si/SiO_2_ NC ensembles. Those can be understood in terms of differences in NC sizes, NC densities and surface quality, without resorting to additional channels of nonradiative recombination^17^.

**Figure 3:**
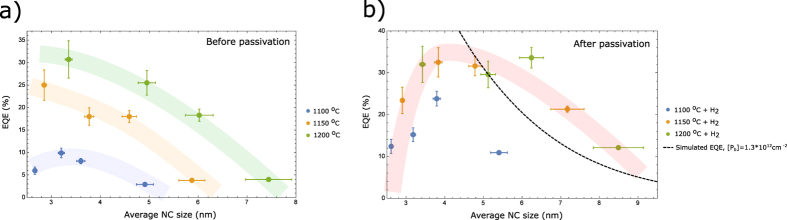
Ensemble emission efficiencies. EQE values at an excitation wavelength of 400 nm, as a function of the average NC size, before passivation (**a**) and after passivation (**b**). The black dotted line in (**b**) depicts the simulated EQE, for an active P_*b*_-defect density of 1.3 * 10^12^ cm^−2^.

In Fig. 3 we depict the EQE values as a function of the average NC size of an ensemble, for all the investigated materials. We note that these efficiencies are the highest ever reported for solid state dispersions of Si NCs within SiO_2_. In the past, EQE values close to 30% have been observed for such samples but only at lower temperatures (around 100 K)^18^, whereas room-temperature values never exceeded 20%^19^,^20^. Three distinct observations can be made:

1. Before passivation, the EQE values crucially depend on the formation temperature (1100–1200 °C).
2. After passivation, the EQE values for samples of the same/similar size converge and therefore become independent of the formation temperature.
3. Independent of the annealing condition, the EQE value peaks for the NC size of around 3–4 nm.

The latter observation is consistent with previous reports^21^,^22^ and can be rationalized, since for larger NCs the surface area increases, which enhances the probability of containing a P_b_-defect^13^. On the other hand, for very small sizes, the increased surface curvature is likely to increase the defect density^2^, and hence the efficiency drops. The first and second observations are coinciding within the proposed quenching model, where higher formation temperatures reduce the P_b_-defect density^23^, which can further be “healed” during the H_2_-passivation process whose kinetics determine the final defect density^9^. From the experimental data, we conclude that the optical performance of sufficiently passivated Si NCs is essentially determined by their size only.

## EQE limit

Assuming that the dominant PL quenching mechanism is facilitated by the P_*b*_-centers, the *P*_*b*_-defect density can be evaluated from the measured EQE values as:
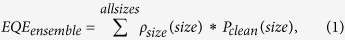
where *ρ*_*size*_ and *P*_*clean*_, refer to the size distribution (here we use a similar size distribution as observed for similar systems^15^) and the probability of producing a defect-free NC, respectively. The latter is given by the Poisson distribution for *n* = 0:
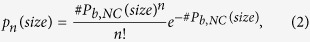
where *n* refers to the amount of *P*_*b*_-defects on the surface of a NC, and #*P*_*b*,*NC*_ expresses the average number of *P*_*b*_-defects per NC:
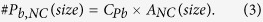


*C*_*Pb*_ and *A*_*NC*_ refer to the surface density of *P*_*b*_-defects and the NC’s surface area, respectively. By taking *C*_*Pb*_ of 1.3 × 10^12^ cm^−2^ we were able to obtain reasonable agreement with the experimental EQE results for NCs above 4 nm – see the black dotted line in Fig. 3. We note that the efficiencies of the surface passivated samples do not significantly exceed this value. Therefore the P_*b*_-defect density stabilizes at this level. The maximum EQE reached in this study is around 35% for Si NC of approximately 4 nm diameter. When converted to an active area defect density, this corresponds to [P_*b*_] ≈ 10^12^ cm^−2^. While under the current approach of material preparation and passivation this level cannot be further reduced, it is still two orders of magnitude higher than what is commonly realized for the planar Si/SiO_2_ interface^23^. If the quality of the planar Si/SiO_2_ interface ([P_*b*_] ≈ 10^10^ cm^−2^) can be reached, PL EQE values close to 100% are certainly feasible for Si NCs. One possible route towards higher efficiencies might be the exploration of passivation with atomic hydrogen, which has more favorable dynamics^24^.

## Conclusions

By using a careful growth technique and H_2_ passivation treatment we were able to develop highly emissive layers of Si NCs in SiO_2_. The highest EQE of ~35% was consistently obtained for NCs with diameters of approximately 3–4 nm, regardless of the particularities of the preparation procedure – silicon excess, and passivation and formation temperatures. We have successfully explained PL efficiency and lifetime within a simple microscopic model where quenching of excitonic PL is induced by P_b_ defects either directly, on a fast timescale for excitation of a defected NC, or indirectly by a slow (microsecond time scale) excitation diffusion towards a defected NC in the neighborhood. Therefore, the optical quality of the Si NCs is determined by a combination of the absolute surface area, the P_b_-defect density, and the distances to neighboring NCs. As a result, we established that the active surface defect density of Si NCs with the highest EQE developed in this study is [P_*b*_] ≈ 1.3 × 10^12^ cm^−2^, which is two orders of magnitude higher than for the state-of-the-art planar Si/SiO_2_ interface. This testifies that further improvement of the optical quality of Si NCs in SiO_2_ towards EQE of 100% is potentially feasible.

## Methods

All thin film Si NC samples were produced by a magnetron co-sputtering method, using high purity Si (99.99%) and SiO_2_ (99.99%) targets. After sputtering, high-temperature heat treatment induces phase separation between Si and SiO_2_, enabling the formation of Si NCs. During preparation both the annealing temperature and the amount of excess Si were varied to create NC ensembles with different sizes^3^. A temperature range of 1100–1200 °C was combined with excess Si amounts ranging from 15 vol% to 25 vol%. Subsequently, a low-temperature annealing at 500 °C within a pure H_2_-atmosphere is performed to passivate the NCs. The procedure consisted of a nitrogen purge to remove oxygen from the tube. Subsequently, the tube was heated to 500 °C. At t = 0 the passivation phase was started: the N_2_ flow was replaced by a H_2_ flow, both at 100 ml/min. At the end of the experiment the oven was opened, while the tube remained sealed, to stimulate rapid cool down, the H_2_ flow was stopped and the tube was purged with N_2_. With this procedure it was found that a maximum effect on the PL EQE occurred already after a passivation phase of around 30 min – the results can be found in the Supplementary Information. Absorption measurements were conducted by making use of a Horiba UV-vis Lambda900 spectrometer. For the time-resolved measurements, a Hamamatsu PMT chip, combined with a Solar M260 monochromator were used. PL EQE measurements were performed in an integrating sphere in order to avoid problems with respect to directionality of reflection and emission. A Xenon lamp coupled to a M130 (Solar LS) monochromator, was used to reach a stable and low excitation intensity regime at a wavelength of 400 nm. All spectra were corrected for the spectral response.

## Additional Information

**How to cite this article**: Limpens, R. *et al.* Emission efficiency limit of Si nanocrystals. *Sci. Rep.* **6**, 19566; doi: 10.1038/srep19566 (2016).

## Supplementary Material

Supplementary Information

## References

[b1] Nozik A. J.. Multiple exciton generation in semiconductor quantum dots. Chem. Phys. Lett. 457(1), 3–11 (2008). 10.1021/jz200166y 26295422

[b2] Dohnalova K., Gregorkiewicz T. & Kusova K.. Silicon quantum dots: surface matters. J. of Phys: Cond. Matt. 26(17), 173201 (2014). 10.1088/0953-8984/26/17/173201 24713583

[b3] Limpens R., Lesage A., Fujii M. & Gregorkiewicz T.. Size confinement of Si nanocrystals in multinanolayer structures. Sc. Rep. 5, 17289 (2015). 26603483 10.1038/srep17289 PMC4658553

[b4] Mangolini L., Jurbergs D., Rogojina E. & Kortshagen U.. Plasma synthesis and liquid-phase surface passivation of brightly luminescent si nanocrystals. J. of lum. 121(2), 327–334 (2006).

[b5] Stesmans A. & Van Gorp G.. Maximum density of Pb_*b*_ centers at the (111) Si/SiO_2_ interface after vacuum anneal. Appl. Phys. Lett. 57(25), 2663–2665 (1990).

[b6] Nishi Y.. Study of silicon-silicon dioxide structure by electron spin resonance. Jap. J. of Appl. Phys. 10(1), 52 (1971).

[b7] Brower K. L.. Kinetics of H_2_ passivation of Pb centers at the (111) Si-SiO_2_ interface. Phys. Rev. B 38(14), 9657 (1988). 10.1103/physrevb.38.9657 9945787

[b8] Poindexter E. H.. Electron paramagnetic resonance studies of interface defects in oxidized silicon. Z. Phys. Chem. 151 (Part_1_2), 165–176 (1987).

[b9] Wilkinson A. R. & Elliman R. G.. Kinetics of h 2 passivation of si nanocrystals in SiO_2_. Phys. Rev. B 68(15), 155302 (2003).

[b10] Takeoka S., Fujii M. & Hayashi S.. Size-dependent photoluminescence from surface-oxidized Si nanocrystals in a weak confinement regime. Phys. Rev. B 62(11), 16820–16825 (2000).

[b11] Cheylan S. & Elliman R. G.. Effect of particle size on the photoluminescence from hydrogen passivated si nanocrystals in SiO_2_. Appl. Phys. Lett. 78(13), 1912–1914 (2001).

[b12] Iacona F., Bongiorno C., Spinella C., Boninelli S. & Priolo F.. Formation and evolution of luminescent Si nanoclusters produced by thermal annealing of SiO_x_ films. J. Appl. Phys. 95(7), 3723–3732 (2004).

[b13] Limpens R. & Gregorkiewicz T.. Spectroscopic investigations of dark Si nanocrystals in SiO_2_ and their role in external quantum efficiency quenching. J. Appl. Phys. 114(7), 074304 (2013).

[b14] Lannoo M., Delerue C. & Allan G.. Theory of radiative and nonradiative transitions for semiconductor nanocrystals. J. Lumin. 70(1), 170–184 (1996).

[b15] Limpens R. *et al.* Resonant energy transfer in si nanocrystal solids. J. of Phys. Chem. C 119(33), 19565–19570 (2015).

[b16] Hiller D., Goetze S. & Zacharias M.. Rapid thermal annealing of size-controlled si nanocrystals: Dependence of interface defect density on thermal budget. J. of Appl. Phys. 109(5), 054308 (2011).

[b17] Sangghaleh F., Sychugov I., Yang Z., Veinot J. G. C. & Linnros J.. Near-unity internal quantum efficiency of luminescent silicon nanocrystals with ligand passivation. ACS nan. 9(7), 7097–7104 (2015). 10.1021/acsnano.5b01717 26083194

[b18] Valenta J., Greben M., Gutsch S., Hiller D. & Zacharias M.. Effects of inter-nanocrystal distance on luminescence quantum yield in ensembles of si nanocrystals. Appl. Phys. Lett. 105(24), 243107 (2014).

[b19] Valenta J.. Determination of absolute quantum yields of luminescing nanomaterials over a broad spectral range: from the integrating sphere theory to the correct methodology. Nanosc. Meth. 3(1), 11–27 (2014).

[b20] Timmerman D., Valenta J., Dohnalová K., De Boer W. D. A. M. & Gregorkiewicz T.. Step-like enhancement of luminescence quantum yield of silicon nanocrystals. Nat. Nanotech. 6(11), 710–713 (2011). 10.1038/nnano.2011.167 21984044

[b21] Sugimoto H., Fujii M., Imakita K., Hayashi S. & Akamatsu. K. Codoping n-and p-type impurities in colloidal silicon nanocrystals: Controlling luminescence energy from below bulk band gap to visible range. J. of Phys. Chem. C 117(22), 11850–11857 (2013).

[b22] Sun W. *et al.* Switching-on quantum size effects in silicon nanocrystals. Adv. Mat. 27(4), 746–749 (2014). 10.1002/adma.201403552 25472530

[b23] Stesmans A.. Structural relaxation of p b defects at the (111) si/sio 2 interface as a function of oxidation temperature: The p b-generation–stress relationship. Phys. Rev. B 48(4), 2418 (1993). 10.1103/physrevb.48.2418 10008634

[b24] Wilkinson A. R. & Elliman R. G.. Maximizing light emission from silicon nanocrystals–the role of hydrogen. Nuc. Instr. and Meth. in Phys. Res. Sect. B: B. Int. with Mat. and At. 242(1), 303–306 (2006).

[b25] Mulloni V., Bellutti P. & Vanzetti L.. Xps and sims investigation on the role of nitrogen in si nanocrystals formation. Surf. Scienc. 585(3), 137–143 (2005).

[b26] Brongersma A. *et al.* Tuning the emission wavelength of si nanocrystals in sio_2_ by oxidation. Appl. Phys. Lett. 72(20), 2577–2579 (1998).

