# Supplementary material for: Emission efficiency limit of Si nanocrystals
Source: Sci Rep. 2016 Jan 20;6:19566. doi: 10.1038/srep19566 (PMC4726123; doi:10.1038/srep19566)
Supplement: Supplementary Information [file srep19566-s1.pdf]

Supplementary Information

To manuscript entitled:

Emission efficiency limit of Si nanocrystals

By Rens Limpens, Stefan Luxembourg, Arthur W. Weeber & Tom Gregorkiewicz.

Several techniques for passivation of Si NCs have been proposed (argon, nitrogen [1], oxygen [2]). While both Ar and N_2 turn out to be relatively inert, hydrogen efficiently passivates the P_b-centers at the NCs' interface [3, 4], coinciding with planar interface physics by Brower [5], and PL intensity can be efficiently increased[6, 7, 4, 8]. Since both the passivation and dissociation of P_b-centers are simultaneously occurring [9, 10], an optimal passivation temperature was determined, being determined by the activation energies of both processes, of around 500 ^oC [7]. Therefore, for the purpose of this study we performed passivation in pure H_2-atmosphere at this specific temperature. In order to be sure that the equilibrium situation is reached, we investigated the ensemble emission efficiency, for a specially prepared sample, as a function of the passivation time (Fig. S.1). As expected, a constant emission efficiency is reached almost immediately, in agreement with previous reports. Since the exact time at which the equilibrium is achieved would probably slightly depend on the NC distribution (determining the NC defect probability), we performed the H_2 passivation for the samples in the study at a duration of 2 hours.

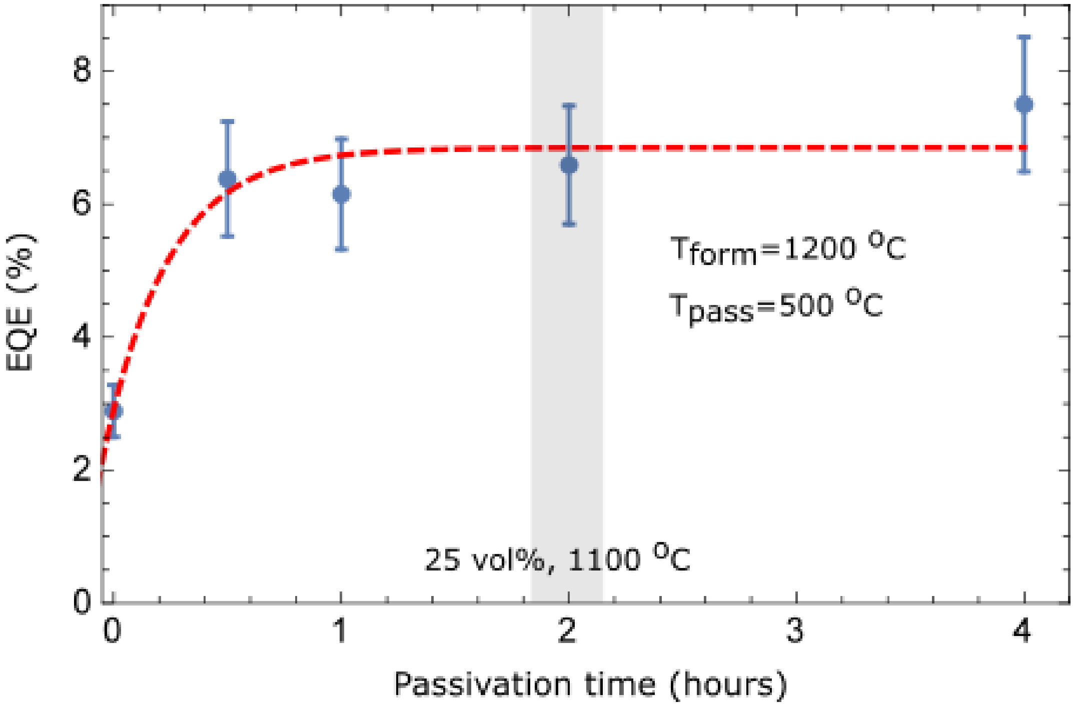

Figure S.1: **Passivation regime.** External quantum efficiency, at an excitation wavelength of 350 nm, as a function of the passivation time at a formation and passivation temperature of 1200°C and 500°C, respectively. A passivation time of 2 hours is used for all the samples in the main paper, as indicated by the grey bar.

References

[1] V. Mulloni, P. Bellutti, and L. Vanzetti. Xps and sims investigation on the role of nitrogen in si nanocrystals formation. *Surf. Scienc.*, 585(3):137–143, 2005.

[2] A. Brongersma, M.L.and Polman, K.S. Min, E. Boer, T. Tambo, and H.A. Atwater. Tuning the emission wavelength of si nanocrystals in sio_2 by oxidation. *Appl. Phys. Lett.*, 72(20):2577–2579, 1998.

[3] K. S. Min, K. V. Shcheglov, C. M. Yang, H. A. Atwater, M. L. Brongersma, and A. Polman. Defect-related versus excitonic visible light emission from ion beam synthesized Si nanocrystals in SiO_2. *Appl. Phys. Lett.*, 69(14):2033–2035, 1996.

[4] A.R. Wilkinson and R.G. Elliman. Maximizing light emission from silicon nanocrystals–the role of hydrogen. *Nuc. Instr. and Meth. in Phys. Res. Sect. B: B. Int. with Mat. and At.*, 242(1):303–306, 2006.

[5] K. L. Brower. Kinetics of H_2 passivation of Pb centers at the (111) Si-SiO_2 interface. *Phys. Rev. B*, 38(14):9657, 1988.

[6] S.P. Withrow, C.W. White, A. Meldrum, J.D. Budai, D.M. Hembree, and J.C. Barbour. Effects of hydrogen in the annealing environment on photoluminescence from si nanoparticles in SiO_2. *J. of Appl. Phys.*, 86(1):396–401, 1999.

[7] A.R. Wilkinson and R.G. Elliman. Kinetics of h 2 passivation of si nanocrystals in SiO_2. *Phys. Rev. B*, 68(15):155302, 2003.

[8] Y. Li, P. Liang, Z. Hu, S. Guo, Q. You, J. Sun, N. Xu, and J. Wu. Enhancement and stability of photoluminescence from si nanocrystals embedded in a SiO_2 matrix by H_2-passivation. *Appl. Surf. Scienc.*, 300:178–183, 2014.

[9] E. Cartier, J.H. Stathis, and D.A. Buchanan. Passivation and depassivation of silicon dangling bonds at the Si/SiO_2 interface by atomic hydrogen. *Appl. Phys. Lett.*, 63(11):1510–1512, 1993.

[10] Y. Jung, J. Yoon, R.G. Elliman, and A.R. Wilkinson. Photoluminescence from si nanocrystals exposed to a hydrogen plasma. *J. of Appl. Phys.*, 104(8):083518, 2008.
